# Prevalence and correlates of vaccine hesitancy among general practitioners: a cross-sectional telephone survey in France, April to July 2014

**DOI:** 10.2807/1560-7917.ES.2016.21.47.30406

**Published:** 2016-11-24

**Authors:** Pierre Verger, Fanny Collange, Lisa Fressard, Aurélie Bocquier, Arnaud Gautier, Céline Pulcini, Jocelyn Raude, Patrick Peretti-Watel

**Affiliations:** 1 INSERM, UMR912 ‘Economics and Social Sciences Applied to Health and Analysis of Medical Information’ (SESSTIM), Marseille, France; 2 ORS PACA, South-eastern Health Regional Observatory, Marseille, France; 3 Aix Marseille Université, UMR_S 912, IRD, Marseille, France; 4 INSERM, F-CRIN, Innovative clinical research network in vaccinology (I-REIVAC), GH Cochin Broca Hôtel Dieu, Paris, France; 5 Aix Marseille University, URMITE, IRD 198, UMR CNRS 7278, INSERM 1095, Faculté de Médecine, Marseille, France; 6 Santé publique France (the French national public health agency), Saint-Maurice, France; 7 CHU de Nancy, Service de Maladies Infectieuses et Tropicales, Hôpitaux de Brabois, Vandœuvre-lès-Nancy, France; 8 Lorraine University, Paris Descartes University, EA 4360 Apemac, Vandœuvre-lès-Nancy, France; 9 EHESP, Sorbonne Paris Cité, Rennes, France; 10 Aix-Marseille University, IRD French Institute of Research for Development, EHESP, UMR_D 190 ‘Emergence des Pathologies Virales’, Marseille, France

**Keywords:** vaccines and immunization, general practice, vaccine hesitancy, attitude of health personnel, behavior, cluster analysis

## Abstract

This article sought to estimate the prevalence of vaccine hesitancy (VH) among French general practitioners (GPs) and to study its demographic, professional and personal correlates. We conducted a cross-sectional telephone survey about GPs’ vaccination-related attitudes and practices in 2014 in a national panel of 1,712 GPs in private practice, randomly selected from an exhaustive database of health professionals in France. A cluster analysis of various dimensions of VH (self-reported vaccine recommendations, perceptions of vaccine risks and usefulness) identified three clusters: 86% of GPs (95% confidence interval (CI): 84–88) were not or only slightly vaccine-hesitant, 11% (95% CI: 9–12) moderately hesitant and 3% (95% CI: 3–4) highly hesitant or opposed to vaccination. GPs in the latter two clusters were less frequently vaccinated and reported occasional practice of alternative medicine more often than those in the first cluster; they also described less experience with vaccine-preventable diseases and more experience with patients who they considered had serious adverse effects from vaccination. This study confirms the presence of VH among French GPs but also suggests that its prevalence is moderate. Given GPs’ central role in vaccination, these results nevertheless call for a mobilisation of stakeholders to address VH among GPs.

## Introduction

Vaccine hesitancy (VH) among lay people is defined as delay in acceptance of vaccination, or refusal of vaccination despite the availability of vaccine services, or even acceptance of vaccination with doubts about its safety and benefits; these behaviours and attitudes vary according to vaccine, personal profile and context (SAGE Group) [1]. VH is also frequently denoted as *'a continuum between those that accept all vaccines with no doubts, to complete refusal with no doubts, with vaccine hesitant individuals the heterogeneous group between these two extremes*’ [2]. VH presents a challenge to physicians, especially to general practitioners (GPs) who are the cornerstone of vaccination implementation in many countries and whose recommendations play an influential role in their patients’ vaccination behaviour [3-5]. In France, GPs write prescriptions for 90% of the vaccinations purchased. Patients may return to the GP for injection after purchasing the vaccine, but they may also see a nurse, make other arrangements or fail to follow up [6].

Although the concept of VH was initially proposed to describe and qualify lack of acceptance of vaccines by lay people, previous publications showed that also physicians report doubts about risks and usefulness of vaccines [7-9] or low vaccine acceptance for themselves [10-12]. Physicians with such doubts may hesitate to recommend vaccination to their patients. We have previously shown that the frequency of French GPs’ self-reported vaccine recommendations for six specific vaccines and target populations (vaccine situations) varied significantly between vaccine situations and GPs [13]. However, because VH is multidimensional (vaccine recommendation behaviour, perceptions of vaccine risks and usefulness) [14], this finding did not allow us to estimate its prevalence directly. Quantifying VH among physicians is essential if public health measures are to be proposed and appropriately scaled to deal with this problem.

This article has two main objectives: (i) to propose a method that can describe and estimate the extent to which GPs hesitate to recommend vaccines to their patients (VH prevalence), taking into account the multidimensional nature of VH, and (ii) to study the demographic, professional and personal correlates of this VH and thus determine whether easily measurable GP characteristics can predict the extent of their VH.

## Methods

### Population

We conducted a cross-sectional telephone survey about vaccination in a national panel of 1,712 GPs in private practice in France. The panel was designed to regularly collect data about GPs’ medical practices and working conditions; the methods used to set it up have been detailed elsewhere [13,15]. In brief, between December 2013 and March 2014, we selected GPs by random sampling from the Ministry of Health’s exhaustive database of health professionals in France. GPs planning to retire within 6 months or who practiced exclusively acupuncture or homoeopathy or other alternative medicine were excluded. Sampling was stratified for sex, age, workload (annual number of office consultations and house calls) in 2012 and the density of each GP’s municipality of practice. The sample size was set so that the smallest stratum contained at least 10 GPs. The panel’s participation acceptance rate was 46% (1,712 of 3,724 eligible GPs that were contacted). The National Authority for Statistical Information (Commission Nationale de l’Information Statistique) approved the panel.

### Procedure and questionnaire

Professional investigators contacted the members of the panel between April and July 2014 to ask them to participate in the survey. They interviewed those who agreed, using computer-assisted telephone interview software and a standardised questionnaire (questionnaire available from the authors on request) [13]. We had developed the questionnaire after reviewing the literature, conducting qualitative interviews with 10 GPs and discussing the questions with a multidisciplinary panel of experts. We had pilot-tested it for clarity, length and face validity among 50 GPs.

Participants were asked about the frequency at which they recommended vaccines in six specific vaccine situations that we had chosen because current coverage does not meet official French objectives. Participants were also asked about their opinions on the likelihood of associations between purported severe adverse effects and certain vaccines or vaccine adjuvants that have been or still are the subject of public or scientific debate in France or elsewhere; they were also asked whether they believed vaccines were useful.

Finally, participants were asked about their professional characteristics, own vaccinations, general opinion about vaccination, perception of their role towards patients in this field, experience of severe side effects potentially related to vaccination (leading to hospital admission) and whether any of their patients in the past five years had had any of the following vaccine-preventable diseases (VPDs): measles, acute or recently diagnosed chronic hepatitis B (HBV), bacterial meningitis, cervical cancer or complicated seasonal influenza requiring hospitalisation. Answers were collected with five-point Likert scales that included a ‘no opinion’ answer for all of these items.

### Statistical analysis

Data were weighted to match the sample more closely to the French national population for stratification variables (sex, age, density of GP’s municipality of practice and workload), taking into account the sampling strategy [13] using SURVEY procedures (PROC SURVEYFREQ, PROC SURVEYLOGISTIC, SAS 9.4 statistical software).

A classification of vaccine-related attitudes and behaviours was developed to estimate VH prevalence among GPs (objective 1) relying on current definitions of VH that make clear that a person’s behaviours and attitudes may vary according to vaccine [2,7]. For that purpose, we performed a multiple correspondence analysis (MCA) combined with an agglomerative hierarchical cluster analysis (AHCA) of the different dimensions of GPs' VH [16]. MCA is an exploratory technique used to understand the inter-relationships between multiple categorical variables [17]; it allows correlated variables to be combined into continuous factors [18]. These factors are introduced in the AHCA, which classifies individuals with similar characteristics into clusters. We used a method based on minimum inertia lost to identify the optimal number of clusters [18,19]; this was defined as a situation when the total within-cluster variance is minimal (individuals with maximum similarity in each cluster) and the between-cluster variance maximal. As VH is also denoted as a continuum between complete refusal of vaccination (radical rejection) and acceptance of vaccines with certainty (ardent support) [2], we also quantified the prevalence of these two extremes among GPs. In this supplementary approach, we defined ‘radical opposition’ by the following criteria: rarely or never recommended vaccines in any of the vaccine situations considered in this study AND reported doubts about usefulness AND risks of vaccines. We defined as ‘ardent supporters’ those GPs who often or always recommended vaccines in all the vaccine situations considered AND did not doubt either usefulness or safety of vaccines.

We used the VH classification as a dependent variable. As we found more than two clusters, we tested their potential correlates (objective 2) with univariable and then multivariable ordered logistic regression models adjusted for stratification variables.

We tested the proportional odds assumption with the score test [20] and computed the variance inflation factor (VIF) to test for multicollinearity between explicative variables [21]. To test whether the differences between panel participants and non-participants might have biased the estimations of the regression analyses, we implemented a bivariate probit model with sample selection. This is a system of two simultaneous equations that make it possible to test for the presence of selection bias and to correct it [22,23]. The first equation was applied to the whole sample of GPs who could be contacted and were eligible (n = 3,724) and analysed the factors associated with participation in the survey using the four stratification variables. The second equation was applied only to GPs who participated in the panel (n = 1,582) and studied the factors associated with the VH classification. Such a model makes it possible to test (and take into account) the correlation (rho) between the error terms that may occur if there are unobservable or unmeasured factors associated with both participation in the survey and vaccine hesitancy, which would bias the estimations; if rho is significant, it is taken into account to calculate unbiased estimates. The likelihood-ratio (LR) test was used to test the null hypothesis of no correlation (rho) between the residuals of these equations.

Finally, we conducted a sensitivity analysis excluding ardent supporters and radical opponents from the multivariable regression. Missing values were excluded from the regression analyses given their limited number.

All analyses were performed in 2015 and based on two-sided p values, with statistical significance defined by p≤0.05; they were conducted with SAS 9.4 statistical software (SAS Institute, Cary, NC).

## Results

In all, 1,712 of 3,724 eligible GPs (46.0%) agreed to participate in the panel. GPs who refused were more often men (p ≤ 10^−3^), older (p ≤ 10^−3^) and had more consultations in 2012 (p ≤ 0.05, data not shown). Two main reasons were reported for refusing: lack of time (55%) and lack of interest in participating in a panel (31%). Of the 1,712 GPs included in the panel, 1,582 (92.4%) participated in the cross-sectional survey; their characteristics did not differ significantly from those of GPs who joined the panel but did not participate in the vaccination survey.

The characteristics of the sample are presented in Table 1. Among the participants, 80% were very and 17% somewhat favourable to vaccination in general, and 90% reported that they would encourage their patients, even those who are reluctant, to be vaccinated. Some 72% reported having had a seasonal influenza shot during winter 2013/14, 84% had had a diphtheria-tetanus-polio (dTPolio) booster in the past 10 years and 86% reported having had three or more doses of vaccine against hepatitis B.

**Table 1:** Characteristics of the study population, nationwide panel of general practitioners, weighted data, France, April to July 2014 (n = 1,582)

	Number	%
*Stratification variables*		
Sex		
Male	1,076	68.0
Female	506	32.0
Age in years (tertiles)		
< 50	538	34.0
50–58	556	35.1
> 58	488	30.8
Density of general practitioners’ municipality of practice (Min–Q1 / Q1–Q3 / Q3–Max)^a^		
< −19.3% of national average	406	25.7
−19.3% to +17.7% of national average	797	50.4
> +17.7% of national average	379	24.0
2012 workload (Min–Q1 / Q1–Q3 / Q3–Max)^a^		
< 3,067 consultations/visits	350	22.1
3,067–6,028 consultations/visits	813	51.4
> 6,028 consultations/visits	419	26.5
*Professional characteristics*		
Practice		
Solo	662	41.9
Group	920	58.1
Coordinator in a retirement home		
No	1,477	93.4
Yes	105	6.6
Work in a healthcare institution		
No	1,315	83.1
Yes	267	16.9
Occasional practice of alternative medicine^b^		
No	1,391	87.9
Yes	191	12.1
Continuing medical education on infectious diseases and vaccination in 2013		
No	899	56.8
Yes	683	43.2
*Practice population characteristics*		
Proportion of patients younger than 16 years (percentage distribution: quartiles)^c^		
0–16	368	25.7
17–21	356	24.8
22–25	368	25.6
26–50	342	23.9
*Experience related to vaccination*		
Has had any patients with at least one vaccine-preventable disease in the past 5 years^d^		
No	169	10.7
Yes	1,413	89.3
Has had any patients with a serious health problem potentially related to vaccination		
No	1,328	83.9
Yes	254	16.1
*Opinions on vaccination in general*		
Favourable to vaccination in general		
Very favourable	1,268	80.2
Somewhat favourable	271	17.1
Not favourable	43	2.7
Perceived role towards patients: convince them to vaccinate, even when they are reluctant		
No	163	10.3
Yes	1,419	89.7
*Personal vaccinations*		
Vaccination against 2013/14 seasonal influenza		
No	449	28.4
Yes	1,133	71.6
Last diphtheria-tetanus-polio (dTPolio) booster		
< 10 years ago	1,325	83.7
10–20 years ago	205	13.0
> 20 years ago	52	3.3
Vaccination against hepatitis B		
Yes, 3 or more doses	1,364	86.2
Yes, fewer than 3 doses	67	4.2
No, or don't remember	151	9.6

^a^ Density of general practitioners' municipality of practice and 2012 workload were categorised so that 25% of GPs were in the first category, 50% were in the second and 25% were in the third category.

^b^ Homoeopathy and/or acupuncture.

^c^ 148 missing values.

^d^ Five vaccine-preventable diseases were mentioned in the questionnaire: measles, acute or recently diagnosed chronic hepatitis B, bacterial meningitis, cervical cancer and complicated seasonal influenza requiring hospitalisation.

Three clusters were identified according to the GPs’ vaccination-related behaviours and perceptions (Table 2). The first cluster (no-to-slight hesitancy) included 86% of GPs (95% confidence interval (CI): 84–88), most of whom considered that vaccines were not at all or not very likely to have severe adverse effects, had no doubts about the usefulness of vaccination and reported recommending vaccination more frequently than the average. The second cluster (moderate hesitancy) included 11% of GPs (95% CI: 9–12): they doubted the safety and usefulness of vaccines more frequently than the average and recommended vaccination less frequently than the sample as a whole. The third cluster (high hesitancy or opposition) included 3% of GPs (95% CI: 3–4%), most of whom considered links between vaccines and severe adverse effects likely or very likely, had doubts about vaccine usefulness, and recommended vaccines much less often than the average.

**Table 2:** Typology of general practitioners according to their practices and opinions about vaccination, agglomerative hierarchical cluster analysis, weighted data, France, April to July 2014 (n = 1,575)

	Vaccine hesitancy (%)			
	No-to-slight n = 1,353 (85.9%)	Moderate n = 166 (10.6%)	High n = 56 (3.5%)	All
*Perceived likelihood of links between specific vaccines and potential severe adverse effects (somewhat/very likely)*				
Seasonal influenza vaccine and Guillain–Barré syndrome	20.1	29.9	66.2	22.8
Hepatitis B vaccine and multiple sclerosis	5.8	30.3	82.8	11.1
Aluminium adjuvants and Alzheimer's disease	5.8	15.2	70.9	9.1
AS03-adjuvanted influenza A(H1N1)pdm09 vaccine Pandemrix and narcolepsy	13.9	28.8	46.4	16.6
Human papilloma virus vaccine and multiple sclerosis	0.2	27.4	50.5	4.8
Vaccines containing adjuvant and long-term complications	24.3	48.2	88.5	29.1
*Perceptions of vaccine* usefulness *(somewhat/strongly agrees)*				
Today some vaccines recommended by authorities are not useful	23.1	40.1	60.4	26.3
Children are vaccinated against too many diseases	16.4	36.5	62.4	20.1
*Frequency of vaccine recommendations (often/always)*				
Measles-mumps-rubella (MMR) to non-immune adolescents and young adults	87.1	55.8	52.6	82.6
Meningococcal meningitis C to 12-month-old infants	70.9	52.8	30.6	67.6
Meningococcal meningitis C to ages 2–24 years (catch-up)	60.6	36.2	20.8	56.6
Human papillomavirus vaccine to girls aged 11–14 years	77.5	46.9	24.5	72.4
Hepatitis B to adolescents (catch-up)	67.1	41.5	29.7	63.1
Seasonal influenza to adults under 65 years with diabetes	87.1	69.9	47.5	83.9

^a^ Seven missing values.

Overall, 85% of GPs in cluster 1, 56% in cluster 2, and 43% in cluster 3 (p < 0.0001) described themselves as very favourable to vaccination in general. Respectively 94%, 73% and 47% (p < 0.0001) agreed that their role is to encourage their patients to be vaccinated, even when patients are reluctant (Table 3).

**Table 3:** General attitudes towards vaccination among the three clusters of general practitioners, weighted data, France, April to July 2014 (n = 1,575^a^)

	Vaccine hesitancy			All	p value^b^
	No-to-slight n = 1,353 (85.9%)	Moderate n = 166 (10.6%)	High n = 56 (3.5%)		
*Attitudes towards vaccination in general*					
Favourable to vaccination in general					
Very favourable	84.7	56.2	43.4	80.3	< 0.0001
Quite favourable	14.5	35.0	24.8	17.0	
Not favourable	0.8	8.9	31.8	2.7	
Perceived role towards patients: convince them to vaccinate, even when they are reluctant					
No	6.5	27.3	52.8	10.3	< 0.0001
Yes	93.5	72.7	47.2	89.7	
Attitude towards vaccination					
Ardent supporter^c^	20.6	7.4	0.0	18.5	< 0.0001
Radical opponent^d^	0.0	1.3	19.0	0.8	
Other	79.4	91.3	81.0	80.7	

^a^ Seven missing values.

^b^ Rao-Scott chi-squared test.

^c^ Frequent recommendations (often/always) in all of the six vaccine situations AND no doubts about vaccine usefulness or safety, excluding items regarding the links between Guillain–Barré syndrome and seasonal influenza and between narcolepsy and Pandemrix, which are evidence-based.

^d^ Rare recommendations (sometimes/never) in all of the six vaccine situations AND doubts about vaccine usefulness and risks, excluding items regarding the links between Guillain–Barré syndrome and seasonal influenza and between narcolepsy and Pandemrix.

In the supplementary continuum approach, 18.5% of GPs were ardent supporters of vaccination (21% in cluster 1, 7% in cluster 2 and 0% in cluster 3), while the proportion of radical opponents was 0.8% (0% in cluster 1, 1% in cluster 2 and 19% in cluster 3; Table 3). Excluding the ardent supporters and radical opponents from the analysis, in accordance with the standard definition of VH, yielded an estimated prevalence of moderate-to-high VH among GPs of 13% (95% CI: 11–14) instead of 14% (95% CI: 12–16) without this exclusion.

The proportional-odds hypothesis was not rejected in the final specified model of the multivariable ordered logistic regression (chi-square (20) = 26.4; p = 0.15). GPs who practiced alternative medicine occasionally, those with no patients who had had one of the five included VPDs, those who had had patients with a serious health problem leading to hospitalisation that might have been related to vaccination as well as those who did not adhere to seasonal influenza or dTPolio vaccine recommendations for themselves, were more prone to moderate-to-high VH in univariable as well as multivariable regressions adjusted for the four stratification variables (Table 4). The test for multicollinearity was negative (VIF < 5). The LR test for the bivariate probit model with sample selection indicated that the estimations of the multivariable regression analysis were unbiased (rho = 0.77; p = 0.42). Exclusion of vaccination supporters and opponents from the analysis produced similar estimates of the odds ratios for the variables of interest (data not shown; results available from the authors on request).

**Table 4:** Factors associated with higher vaccine hesitancy among general practitioners', ordered logistic regressions, weighted data, France, April to July 2014 (n = 1,427^a^)

	Univariable regression	Multivariable regression
	Odds ratio (95% CI)	Adjusted odds ratio (95% CI)
*Stratification variables*		
Sex (ref. Male)		
Female	0.92 (0.69–1.23)	0.94 (0.63–1.38)
Age in years (ref. < 50)		
50–58	1.12 (0.79–1.59)	0.67 (0.44–1.03)
> 58	**1.69 (1.19–2.38)**	1.00 (0.63–1.61)
Density of GP’s municipality of practice (ref. < −19.3% of national average)		
Between −19.3% and +17.7% of national average	0.77 (0.55–1.07)	0.76 (0.52–1.11)
> +17.7% of national average	1.09 (0.76–1.58)	1.09 (0.72–1.66)
2012 workload (ref. < 3,067 consultations/visits)		
3,067–6,028 consultations/visits	**0.39 (0.28–0.55)**	0.69 (0.46–1.04)
> 6,028 consultations/visits	**0.50 (0.35–0.72)**	0.91 (0.58–1.45)
*Professional characteristics*		
Practice (ref. Solo)		
Group	**0.59 (0.45–0.79)**	1.10 (0.77–1.57)
Coordinator in a retirement home (ref. No)		
Yes	0.67 (0.35–1.28)	0.92 (0.45–1.89)
Work in a healthcare institution (ref. No)		
Yes	0.67 (0.44–1.02)	0.74 (0.45–1.21)
Occasional practice of alternative medicine^b^ (ref. No)		
Yes	**5.68 (4.04–7.98)**	**2.89 (1.94–4.31)**
Continuing medical education on infectious diseases and vaccination in 2013 (ref. No)		
Yes	**0.65 (0.49–0.88)**	0.94 (0.67–1.32)
*Characteristics of practice population*		
Proportion of patients aged under 16 (0–50%)	**0.97 (0.95–0.99)**	0.99 (0.96–1.01)
*Experience related to vaccination*		
Number of different vaccine-preventable diseases among the GP’s patients (0–5)^c^	**0.70 (0.62–0.80)**	**0.78 (0.67–0.90)**
Has had patients with a serious health problem potentially related to vaccination (ref. No)		
Yes	**2.30 (1.64–3.22)**	**1.82 (1.23–2.68)**
*Personal vaccinations*		
Vaccination against 2013–2014 seasonal influenza (ref. Yes)		
No	**4.48 (3.34–6.01)**	**2.51 (1.78–3.54)**
Last diphtheria-tetanus-polio (dTPolio) booster (ref. <10 years ago)		
10–20 years ago	**2.37 (1.63–3.43)**	**1.58 (1.02–2.46)**
>20 years ago	**6.60 (3.60–12.08)**	**2.23 (1.16–4.26)**
Vaccination against hepatitis B (ref. Yes, 3 or more doses)		
Yes, fewer than 3 doses	**2.76 (1.55–4.89)**	1.36 (0.72–2.57)
No, or don't remember	**4.22 (2.87–6.21)**	1.55 (0.94–2.55)
Nagelkerke R^2^		0.21

CI: confidence interval; GP: general practitioner.

^a^ 155 GPSs were excluded because of missing values about the characteristics of their practice population (n = 148) or about their vaccine hesitancy (n = 7).

^b^ Homoeopathy and/or acupuncture.

^c^ Five vaccine-preventable diseases were mentioned in the questionnaire: measles, acute or recently diagnosed chronic hepatitis B, bacterial meningitis, cervical cancer and complicated seasonal influenza requiring hospitalisation.

## Discussion

The prevalence of moderate-to-high VH was 14%. Compared with those with no-to-slight VH, GPs with moderate-to-high VH were less frequently vaccinated, reported more often that they occasionally practiced alternative medicine, and reported fewer patients with VPDs and more with serious adverse effects possibly due to vaccination.

Despite the moderate prevalence of VH among GPs, our results are worrying because GPs play an essential role in vaccinating their patients, answering their questions and addressing their VH (a growing phenomenon in the general population [24]). Evidence indicates that most parents seek information and advice from their healthcare provider regarding VPDs, vaccines and the recommended vaccination schedule [25]. GPs with moderate-to-high VH were less prone to try to convince hesitant patients to be vaccinated (or have their children vaccinated). A previous publication showed a strong positive association between the frequency of GPs’ recommendations for various vaccines and their self-perceived efficacy in explaining the benefits and risks of vaccines to their patients [13]. Given the strong influence of GPs on their patients’ vaccination decisions [3-5], their VH may impede efforts to alleviate patients’ VH.

The strong association between occasional practice of alternative medicine and moderate-to-high VH was expected: physicians belonging to this category were those who occasionally practiced homoeopathy or acupuncture; they accounted for 12% of GPs [26] in France in 2010. These GPs vaccinate themselves less frequently than other GPs (e.g. against hepatitis B and pandemic and seasonal influenza [27]) and are frequently less favourable to vaccination than other physicians [13]. Previous studies showed reduced adherence to paediatric vaccination schedules and reduced acceptance of vaccines in their patients [28].

Our results suggest that GPs’ experience of both VPD and adverse effects of vaccines may influence their VH level more than their academic education in infectious diseases and vaccination. GPs with moderate-to-high hesitancy may perceive that adverse effects are more common than those with no-to-slight hesitancy. Our results are also consistent with previous publications that found that GPs’ knowledge from their own individual practice experience and from the Internet, the media and patients might be more influential than academic and technical knowledge in shaping GPs’ perceptions of the risk/benefit balance of vaccines, especially in controversial situations [29]. This could be explained in part by the major gaps identified in Europe, including France, in the initial training and continuous medical education of physicians regarding vaccination, by the difficulties in keeping them informed during continuously evolving situations and, in some cases, by feelings of distrust towards health institutions [13,30].

Surprisingly, doubts existed about vaccine risks even among the numerous GPs with no-or-slight VH. This remained true even after excluding answers about the two evidence-based risks: Guillain–Barré syndrome after seasonal influenza vaccination and narcolepsy after Pandemrix vaccination [31]. The safety of vaccines and adjuvants has been the subject of persistent controversy in France since the 1990s. While French GPs do not consider the media to be a reliable source of information in the field of vaccination [13], the media’s role in setting the risk agenda and its amplification of controversial positions may affect perceptions of vaccine risks in GPs as much as it does in the lay population. Observing these doubts among the least hesitant GPs, most of whom were very favourable to vaccination in general, shows how fragile their pro-vaccination attitudes may be.

The fact that a quarter of the least hesitant GPs thought that some vaccines recommended by French health authorities are not useful is also surprising. Doubts among physicians about the usefulness of vaccines have been observed in studies throughout the world [7]; some doctors consider that certain VPDs are too infrequent to justify systematic vaccination, a perception shared by some French GPs, in particular for meningitis C and hepatitis B [32]. These perceptions may also reflect the opinion that the official French vaccine schedule is becoming increasingly complex: constant change to the schedule makes it difficult for doctors to adapt and may adversely affect patient acceptance [32].

### Limitations

By joining the panel, GPs agreed to take part in five different surveys during a 30-month period: the good participation rate (46%) compared with other primary physician panels (for example the panel in Joyce et al. (2010) with a response rate of 19.4% [33]), does not rule out the possibility of selection bias. In particular, panel participants and non-participants differed in age, sex and workload [13]. Nonetheless, we weighted the sample according to these variables, which should have corrected a potential selection bias. Moreover, to limit potential selection bias related to particular attitudes about vaccination, the specific topic of the surveys was not mentioned to GPs when they were first invited to participate in the panel. In the overall panel, participants in the vaccination survey did not differ from non-participants. Finally, the results of the bivariate probit model indicated that the regression parameters of the multivariable model were unbiased.

GPs’ vaccine recommendation behaviour was self-reported, which is a limitation that our study shares with previous publications: declaration or desirability biases cannot be excluded. However, questionnaire data appears to overestimate vaccination rates by less than 10% [34] and self-reported vaccination coverage (e.g. for pandemic or seasonal influenza) in hospital healthcare workers has been shown to be a good proxy for recorded vaccine coverage [35]. In any case, our study’s aim was not to estimate vaccine coverage among GPs but the prevalence of VH among them. GPs’ self-reported recommendations are useful indicators for that purpose because (i) they reflect in part the degree to which GPs are favourable to vaccines and (ii) retrieving reliable information about GPs’ recommendation behaviour from patients' files was not feasible [13]. In addition, this questionnaire method is easily reproducible and could be used to monitor trends in VH over time for GPs.

Because this vaccination survey was cross-sectional and retrospective, no causal inferences can be drawn. Finally, we may not be able to extrapolate our results directly to other countries where VH is likely to exist among healthcare workers [36] because some of the vaccine situations we addressed in this study are specific mainly to France.

The approach used in this article allowed us to estimate VH prevalence among GPs while taking its multidimensional nature into account. The resulting VH typology appears robust: it was strongly correlated with the GPs’ own vaccination behaviour and with their opinion towards vaccination in general. That approach can be applied elsewhere, although the vaccines and target populations chosen would probably differ from those selected here.

## Conclusions

Our results underline the need to better coordinate the mobilisation efforts by public health institutions and other actors to address VH among GPs in France. Efforts should be directed with priority to GPs with moderate-to-high VH. Nonetheless, efforts to inform and support GPs with no-to-low VH are also warranted to prevent some of the existing reservations and the expansion of VH in this group.

Improving GPs’ knowledge of vaccination and vaccines is a necessary but not sufficient condition to modify their behaviours and attitudes in this area [36]. This should lead health authorities to promote and evaluate multicomponent interventions including in particular education, individualised feedback and strong quality incentives, all of which have proven to be effective strategies [37]. Given the variation of VH intensity between GPs, tailored interventions taking GPs’ baseline VH level into account should be tested. More research is needed to quantify and monitor VH in different medical occupations and in different countries.
